# Scraping Spoon

**Published:** 1878-09-20

**Authors:** 


### SCRAPING-SPOON

(Mr. Balmanno Squire's)

FOR THE TREATMENT OF LUPUS VULGARIS, LUPUS ERYTHEMATOSUS, RODENT ULCER (FLAT EPITHELIAL CANCER), SCROFULOUS ULCERS, AND SOME OTHER CIRCUMSCRIBED CHRONIC LESIONS OF THE SKIN.

*Explanation.*—The application of the treatment by erasion to certain chronic, progressively ulcerating lesions of the skin was originated by Volkmann, of Halle, in its application to lupus vulgaris. The use of this method has been recently extended to some other skin diseases, and has met with the warm approval of such authoiities as Auspitz and Hebra, of Vienna. The result attained by erasion is the mechanical removal of such diseased deposits in the skin as, having once obtained a *locus standi*, exhibit but little, if any, tendency to spontaneous involution, and are with difficulty, if at all, remediable by any other than such heroic measures as complete excision or deep cauterization. The constant tendency of such deposits as are here referred to is either by slow progress to extend the area of their occupation of the skin, or by ulceration of their morbid structure to lead to more or less extensive destruction of the skin, and, as in some instances, of the subjacent tissues also.

It has been found, as a matter of practical trial, that the removal of the diseased deposit by the simple method of scraping it away is, in many lesions, such as those above enumerated, quite sufficient to convert the diseased area into a healthy ulcer, which thereupon speedily heals up (namely, within from a week to a month, according to its extent), and so the cure of an obstinate and long-standing disease of months' or even years' duration may often thus be rapidly accomplished. The employment of this method is especially facilitated by the circumstance that, in all of the lesions above particularized, the diseased structure is so extremely friable that the readiness with which it crumples away under the pressure of the spoon at once differentiates it to the sense of touch (as conveyed by the spoon) from the notably tough surrounding healthy textures, namely, from the circumjacent sound skin, and from the subjacent cellular tissue, so that the sense of touch materially aids the eye in distinguishing the unsound from the sound structure. In point of fact, it is impossible to remove any of the sound structures by means of this instrument if the instrument be used with only moderate force; the extremely tough character of healthy fibrous tissue effectively precludes such a contingency.

One considerable advantage of this method is its absolutely complete conservation of the neighboring sound textures, which, if only moderately firm pressure be exerted in using the spoon, are left quite intact. In this respect, the method of erasion contrasts favorably with either excision or deep cauterization, since, in either of the latter procedures, a more or less considerable portion of the surrounding sound structure is either removed or hopelessly destroyed. This consideration is of special importance, inasmuch as, in diseases such as those in question, which are especially apt to attack the face or the neck, and which are formidable chiefly on account of the extensive disfiguration they are wont to lead to, it becomes a matter of consequence to refrain, as far as possible, from adding to the loss of substance already produced by the disease by occasioning a further loss of substance by the interference necessary to the cure of the disease.

Mr. Squire's instruments are modifications of the instruments in use in Germany. The advantages presented by the English instruments are that they are smaller, deeper, and narrower than the German spoons.

*Mode of Operating.*—It is unnecessary to chloroform the patient. Local anæsthesia, produced by means of the ether-spray, amply suffices to prevent the operation being felt by the patient. Immediately before operating, the skin should be well frozen by the spray; but inasmuch as the *frozen* skin presents no distinction as to where sound and where not sound to the touch of the spoon, because both healthy and diseased structures are equally hard when frozen, it is therefore necessary to allow the skin to begin to thaw before operating. Immediately that the chilled surface is noticed to have lost the characteristic tallowy whiteness of frozen skin, the scraping should be expeditiously performed. If the patch be but of moderate size, and the operator fairly adroit, the operation may be neatly completed without the patient at all feeling it. All bleeding may be at once arrested by exercising moderately firm pressure on the scraped surface with the finger tips, a layer of blotting paper being interposed between them and the raw surface.

The instrument should be held slantingly in the manner of a writing pen or a drawing pencil, and the movements of the hand, in using it, are such as are suggested by this remark. It is especially necessary that the edges of the patches should be scraped thoroughly, since it is at the margin of the patches that the disease commonly exerts its greatest activity.

Small outlying nodules or specks of morbid deposit which may be found (for example, often in cases of lupus vulgaris) around the main patches of the disease, are to be dealt with in a somewhat different manner, namely, by holding the instrument perpendicular to the skin, with the end of the spoon resting on the nodule. The tip of the forefinger of the left hand is then to be placed on the butt-end of the instrument, so as to steady it in its position, and at the same time to press it with moderate firmness against the skin. At the same time, the handle of the instrument is to be rotated in one direction continuously by means of the thumb and forefinger of the right hand. After two or three turns of the instrument, the nodule will be found to be completely enucleated.

As to the after-treatment, nothing more is necessary than for the first three or four days to smear a thin film of simple ointment over the wound, and subsequently a film of weak resin ointment, increasing progressively the strength of the resin ointment if need be. It is an advantage to wash the wound regularly once, or even thrice a day with warm soap and water, applied by means of a large camel-hair brush, so as to cleanse it thoroughly from discharge before each fresh application of ointment.

